# Editorial: Postural control priorities and effective motor learning

**DOI:** 10.3389/fnhum.2024.1542432

**Published:** 2025-01-09

**Authors:** Tadayoshi Asaka, Yoshiro Okubo, Naoya Hasegawa

**Affiliations:** ^1^Hokkaido University, Sapporo, Hokkaido, Japan; ^2^Neuroscience Research Australia, Sydney, NSW, Australia; ^3^Faculty of Health Sciences, Hokkaido University, Sapporo, Hokkaido, Japan

**Keywords:** postural control, motor learning, priorities, postural balance, motor control

## Introduction

Postural control is the mature ability to establish or restore one's postural balance in any position or during any motor activity. Previous studies on postural control and postural motor learning have elucidated how the central nervous system acquires, adjusts, and learns postural behaviors. However, the interpretation of these controlled behaviors presents some challenges when incongruent postural control priorities exist. These include but not limited to trade-offs between stability and flexibility, automatic vs. voluntary control, and inherent vs. compensatory mechanisms. This involves reliance on certain sensory domains such as visual, vestibular, proprioceptive or tactile feedback when adapting its posture to environmental stimuli. A better understanding of postural control priorities can aid in finding an effective and personalized approach to motor learning in healthy younger and older adults, rehabilitation for individuals with neurological diseases, and promote healthy daily living.

Research to understand postural control priorities—that is, individual's preference for postural control matured by the adaptation to their environment—takes various forms. For example, Kluzik et al. (2005) reported that approximately half of the participants showed an inclined postural alignment after standing on a tilting board when they returned to standing on the horizontal surface, while the others can maintain a vertical postural alignment. Such post-incline learning after-effect suggests a preferential reliance on proprioceptive somatosensory information over graviceptive somatosensory or vestibular information for postural orientation. Therefore, the effective learning approach for postural orientation could be different between the learners and non-learners. The deeper interpretation of the controlled behaviors focused on the postural control priorities and a more effective learning approach based on that interpretation are addressed in this Research Topic. The objective of this Research Topic is two: (1) To establish an openly available resource highlighting current evidence on postural control priorities, and (2) to guide future investigations aimed at elucidating the impact of postural control priorities on effective motor learning. Multidisciplinary approaches including biomedical engineering, neurophysiology, and computational neuroscience are essential to enhance the understanding of postural control priorities and their impact on motor learning. This Research Topic thus aims to expand the discussion of recent advances, technologies, solutions, applications, and emerging challenges in the fields of motor control and motor learning.

## Highlights from the Research Topic

- Smoothness in stand-to-sit movement: Jeon et al. investigated how smoothness and speed of stand-to-sit movements influence joint kinematics, kinetics, muscle activation patterns, and postural stability in younger adults. Their findings highlight how smooth stand-to-sit movements facilitate enhanced control of hip and knee joints, falling momentum and reduced postural sway, indicating the importance of smoothness in transitional movements. The study results also imply two groups of participants with low and high variabilities of center-of-mass (CoM) acceleration during the stand-to-sit task (Figure 4C), which may suggest different postural control priorities that should be accounted for in a training program.
- Facial expressions and stepping reactions: Lebert et al. explored the influence of facial expressions (anger or fear) and gaze (direct or averted) on stepping and quiet standing in younger adults. The findings suggested faster step initiation with angry faces but no significant effect of gaze, implying impacts of emotional stimuli on postural responses. Regarding individual preference, the reaction time in response to fearful face could be divided into two groups especially in associated with an averted gaze (Figure 4). Identifying the factors that distinguish these groups could provide deeper insights into individual variability in postural control strategies.
- Mental vs. physical training in motor learning: Sato examined the effects of mental and physical training on task generalization and retention in a drawing task in younger adults. Mental training produced greater immediate generalization, regardless of task difficulty, reflecting a prioritization of cognitive strategies in motor learning. However, generalization of an easier task was more effectively retained than more difficult tasks. The findings provide insights into trade-offs between cognitive and physical components as well as easy and difficult tasks in effective motor learning.
- Tai Chi practice and sensory reweighting: Liu et al. investigated how long-term Tai Chi practice affects sensory reweighting and self-motion perception under multisensory perturbations in older adults. Tai Chi practitioners exhibited higher postural stability, higher complexity of postural fluctuations, greater weighting on unperturbed sensory systems and faster recovery from disturbances. These findings illustrate how sensory prioritization may be improved with long-term exercise training that challenges postural controls.

## Conclusion

Although the Research Topic concluded with fewer manuscripts than initially planned, the contributions provide meaningful advancements in understanding postural control priorities and motor learning. Each study offers valuable insights into specific factors—such as movement smoothness, emotional stimuli, gaze direction, and training modalities—that shape balance and motor learning outcomes. Moreover, the significant individual variability reported across studies underscores the potential to further analyze these differences to identify distinct postural control priorities, paving the way for personalized training programs. The findings from this initiative highlight its critical importance, and we remain optimistic that it will spark further research and drive progress in the fields of motor control and motor learning.

## References

[B1] Kluzik J. Horak F. Peterka R. (2005). Differences in preferred reference frames for postural orientation shown by after-effects of stance on an inclined surface, Exp. Brain Res. 162, 474–489. 10.1007/s00221-004-2124-6 15654594

